# Preloading budding yeast with all-in-one CRISPR/Cas9 vectors for easy and high-efficient genome editing

**DOI:** 10.14440/jbm.2018.254

**Published:** 2018-09-12

**Authors:** Daniel Degreif, Milana Kremenovic, Thomas Geiger, Adam Bertl

**Affiliations:** Technische Universität Darmstadt, Department of Biology, Yeast Membrane Biology, Darmstadt, Germany

**Keywords:** all-in-one, CRISPR/Cas9, inducible, mating type switching, multiplex genome editing, *Saccharomyces cerevisiae*

## Abstract

The CRISPR/Cas9 technology has greatly improved genome editing in *Saccharomyces cerevisiae* over recent years. However, several current CRISPR/Cas9 systems suffer from work-intensive cloning procedures and/or the requirement of co-transforming target cells with multiple system components simultaneously which can reduce the effectivity of such applications. Here, we present a new set of all-in-one CRISPR/Cas9 vectors that combine unique benefits of different already existent systems in order to further expand the technology’s design possibilities. Our vectors mediate constitutive gRNA expression whereas Cas9 expression is either driven from a constitutive or an inducible promoter. The introduction of desired gRNA targeting sequences into our inducible single gRNA vector relies just on *in vivo* homologous recombination-mediated assembly of overlapping single-stranded oligonucleotides, thus reducing efforts of plasmid cloning to an absolute minimum. By employing the inducible system, yeast cells can be easily preloaded with plasmids encoding for a functional CRISPR/Cas9 system, thereby chronologically separating the cloning procedure from the genome editing step. Gene knockouts could be achieved with high efficiency and effectivity by simply transforming preloaded cells with a selectable disruption cassette without the need of co-introducing any CRISPR/Cas9 system component. We also show the feasibility of efficient gene knockouts even when multiple gene copies were present such as in non-haploid strain backgrounds as well as the simultaneous deletion of two different genes in a haploid genetic background by using a multiplex variant of our inducible vector. The versatile applicability of our inducible vector system was further demonstrated by CRISPR/Cas9-mediated mating type switching of yeast.

## INTRODUCTION

The CRISPR/Cas9 technology has become an important and powerful tool in yeast genome editing and is by now a routinely used biomolecular technique in yeast laboratories all over the world. Daily routine benefits from a fast and affordable technique that is additionally easy in application. Thus, the “yeast community” has developed a number of variants of the CRISPR/Cas9 technology that allow for a convenient application of the system [1].

A functional CRISPR/Cas9 system consists of two components—the Cas9 endonuclease and a guide RNA (gRNA). The Cas9 protein binds the gRNA, which contains a 5’-terminal 20 bp targeting sequence, to form a functional protein-RNA complex. By this means, Cas9 is enabled to scan DNA targets such as genomic DNA in a gRNA-guided manner for sequences (so-called protospacer sequences) that are identical to the specific gRNA-targeting sequence. Identified target sequences are cleaved by the Cas9 endonuclease if a protospacer adjacent motif (PAM) follows the protospacer sequence within the genome. Early plasmid-based approaches employed separate vectors for expressing Cas9 and the gRNA. Thereby, gRNA cassette-encoding plasmids had to be cloned every time from scratch to target a new gene locus, resulting in an inconvenient overall procedure. Consecutive transformations of the Cas9 expression plasmid and the gRNA-encoding plasmid (plus donor DNA where applicable) were necessary to yield efficient gene disruption or genomic integration of the donor DNA since the overall efficiency suffers from the multiplicative linking of the efficiency of the individual transformation events. These restrictions could be avoided by stably integrating a Cas9 expression cassette into the genome so that only the gRNA has to be expressed from an episomal plasmid [2]. This way, genetic modifications can be performed efficiently by just co-transforming a respective gRNA-encoding plasmid together with the donor DNA. Nevertheless, this approach requires previous time-consuming genetic modifications of the desired background strain which also limits its simple use.

Recently, various approaches were published that allow for a simultaneous expression of Cas9 and the gRNA from a single plasmid as well as convenient introduction of the 20 bp gRNA protospacer sequence. These were either introduced by restriction free cloning [3], PCR-based amplification of the whole vector backbone combined with Gibson Assembly [4] or *in vitro* ligation [5], Gibson Assembly mediated introduction of oligonucleotides [6], Golden Gate Assembly with protospacer sequence containing gBlocks [7] or by classical restriction ligation-based cloning using hybridized oligonucleotides as an insert [8]. One-step transformation of all CRISPR/Cas9 system components together with the donor DNA yielded genomic editing in all cases. Although these approaches already allow for an easy application of the CRISPR/Cas9 technology, we were seeking for further streamlining the workflow by simultaneously providing new design possibilities of vector based CRISPR/Cas9-approaches in *Saccharomyces cerevisiae* (*S. cerevisiae*). One improvement was inspired by the “synthesis of DNA fragments in yeast by one-step assembly of overlapping oligonucleotides” [9] which should be an appropriate method for a straightforward introduction of the 20 bp protospacer sequence into the expression vector. By using this method, plasmid cloning relies solely on the robust yeast-endogenous homologous-recombination (HR) machinery without the need for any PCR applications or any other conventional *in vitro* cloning method. A second improvement should help to overcome limitations of transformation efficiency that result from co-transforming of multiple system components (donor DNA and gRNA/Cas9 encoding elements) which is an essential step of all so far published CRISPR/Cas9 technology variants. In our system, we introduce the gRNA protospacer sequence into the plasmid by *in vivo* recombination-based cloning and use yeast cells derived from a single clone for a second transformation with the respective donor DNA. Thus, every single yeast cell used for this second transformation should be already preloaded with all essential CRISPR/Cas9 components and the overall efficiency relies solely on the transformation efficiency regarding the donor DNA and is not a multiplicative product of single transformation efficiencies of different system components. By exploiting the tightly controllable yeast endogenous *GAL1* promoter for expression of the Cas9 endonuclease, the CRISPR/Cas9 system is kept inactive until donor DNA is delivered and intracellularly available, thus preventing induction of DSBs without proper repair using donor DNA as a template for homology directed repair.

## MATERIALS AND METHODS

### Yeast strains and plasmid construction

Experiments were performed with the haploid yeast strains BY4741 [10], CEN.PK2-1C and CEN.PK2-1D [11], the diploid strain CEN.PK2 [11] and the tetraploid yeast strain YCC78 (Y558) [12]. CEN.PK2 strains and BY4741 were obtained from EUROSCARF. Detailed information on the respective genotypes is available in **Table 1**.

Wild-type strains were routinely cultured in YPD media (2% (w/v) glucose, 2% (w/v) peptone, 1% (w/v) yeast extract). Strains were grown in appropriate synthetic defined drop-out media (2% (w/v) glucose or 2% (w/v) galactose, yeast nitrogen base (YNB), amino acid supplement) for plasmid maintenance or for selection of prototrophic phenotypes. Cells were routinely propagated at 30°C.

Detailed information on plasmid construction is provided in the supplemental information. Plasmids are available on request.

### gRNA protospacer sequences

The anti-*ADE2* protospacer sequence (AATTGTAGAGACTATCCACA) was taken from the previously described CasEMBLR approach [14]. The anti-*CAN1* protospacer sequence (GATACGTTCTCTATGGAGGA) was adopted from DiCarlo *et al*. [15]. The protospacer sequences anti-*ADE8* (GAGAACAAGCCTCTGACGGC), anti-*MATx* (TCTTCTGTTGTTA CACTCTC) and anti-*MATz* (CACTCTACAAAACCAAAACC) were designed by using the ATUM gRNA Design Tool (https://www.atum.bio/eCommerce/cas9/input).

### Introduction of gRNA protospacer sequences into plasmids pCAS9c(d) and pCAS9i(d)

Three different methods were used to introduce the respective gRNA protospacer sequence(s) into our all-in-one CRISPR/Cas9 plasmids pCAS9c (constitutive), pCAS9i (inducible); pCAS9cd (constitutive; duplex) and pCAS9id (inducible; duplex).

Method A: Co-transformation of KpnI & PmeI (NEB) cleaved plasmid pCAS9c/pCAS9i (250 ng) with oligonucleotides (500 pmol each; P12 & P13 for *ADE2*; P21 & P22 for *ADE8*) carrying the protospacer sequence or the complementary sequence, respectively, at their 5’ ends.

Method B: Co-transformation of KpnI & PmeI (NEB) cleaved plasmid pCAS9c/pCAS9i (250 ng) with the anti-*ADE2* gRNA expression cassette (10 µl) generated *via* assembly PCR.

Method C: The entire vector backbone was amplified (Q5 DNA polymerase, NEB) with primers carrying the protospacer sequence or the complementary sequence, respectively, at their 5’ ends (P12 & P13 for *ADE2*; P21 & P22 for *ADE8*) using KpnI & PmeI cleaved pCAS9c/pCAS9i as the template. Twenty µl of the unpurified PCR product were directly used for transformation. In case of pCAS9cd/pCAS9id, the plasmids were amplified by two individual PCRs with overhang primers adding different gRNA protospacer sequences to the ends of each plasmid fragment (P12 & P28 for the large fragment and P13 & P29 for the short fragment of pCAS9cd/pCAS9id-anti*ADE2*/*CAN1*; P34 & P35 for the large fragment and P33 & P36 for the short fragment of pCAS9id-anti*MATx*/*MATz*). The large fragments were amplified from NotI-cleaved template plasmids whereas StuI & SalI cleaved plasmids served as templates for amplifying the short fragments. Ten µl of each PCR product were used for co-transformation.

Circularization of the plasmids should be achieved for all methods *via* homologous recombination in yeast without requiring any other *in vitro* cloning method. This keeps the systems as simple as possible.

As a proof of principle, successful introduction of the *ADE2* targeting protospacer sequence into plasmid pCAS9i with methods A, B or C was proved with colony PCR. A pair of primers was used with one primer (P17) binding within the protospacer sequence and the other one (P18) within the plasmid backbone.

Primers and oligonucleotides used in this study are listed in **Table S1**.

### Assays for testing functionality and efficiency of pCAS9c- and pCAS9i-based approaches

For testing plasmid pCAS9c, yeast cells were inoculated at OD_600_ = 0.2–0.3 in YPD and grown to OD_600_ = 0.9–1.0 at 30°C with continuous shaking (200 rpm). Cells from 4 ml of this suspension were used for transformation using the Frozen-EZ Yeast Transformation II kit (Zymo Research). Depending on the method (A, B or C) employed for introducing the gRNA protospacer sequence, cells were transformed with the respective DNA components and PCR-amplified donor DNA (loxP-*LEU2*-loxP for *ADE2* locus: 1 µg (20 µl); loxP-kanMX-loxP for *ADE8* locus: 1 µg (20 µl); *HIS3* for *CAN1*: 1 µg (20 µl)).

The respective gRNA targeting sequence was introduced in plasmid pCAS9i using method A (for pCAS9id with method C) and cells harboring pCAS9i with introduced gRNA targeting sequence were selected on SD-ura agar plates. Single colonies were inoculated in SD-ura and grown over night at 30°C. From the overnight cultures, fresh cultures were set up at OD_600_ = 0.2–0.3 in SD-ura and grown to OD_600_ = 0.9–1.0 at 30°C with continuous shaking (200 rpm). Cells from 4 ml suspension were washed once with sterile water, transferred to 4 ml SGal-ura medium and incubated for 1 h at 30°C. These cells were transformed with PCR-amplified donor DNA (see above) using the Frozen-EZ Yeast Transformation II kit (Zymo Research).

To enhance transformation efficiency, cells were resuspended in 1 ml YPD (pCAS9c) or YPGal (pCAS9i) after incubation in EZ3 solution (1 h) and incubated at 30°C for 2 h for recovery. After the recovery period, cells transformed with loxP-*LEU2*-loxP donor DNA were spread on SD-ura-leu or SGal-ura-leu agar plates, respectively. YCC78 cells that were transformed with loxP-kanMX-loxP donor DNA were plated on YPD+G418 (200 µg/ml) or YPGal+G418, respectively, to select for positive transformants. G418 resistant transformants were replica plated on SD-ura plates to screen for the presence of pCAS9 plasmids. Cells that were disrupted in the *CAN1* locus were selected in arginine-free (-arg) canavanine-containing (60 µg/L) medium.

For gRNA negative control experiments (-gRNA), unmodified plasmids pCAS9c or pCAS9i (250 ng) harboring no proper gRNA targeting sequence were employed for previously described approaches.

loxP-*LEU2*-loxP donor DNA for integration into the *ADE2* locus was amplified from plasmid pUG73 [16] by PCR (OneTaq DNA polymerase, NEB) using primers P15 and P16. loxP-kanMX-loxP donor DNA for integration into the *ADE8* locus was amplified from plasmid pUG6 [17] by PCR (OneTaq DNA polymerase, NEB) using primers P23 and P24. *HIS3* donor DNA for integration into the *CAN1* locus was amplified from plasmid pGREG504 [18] by PCR (OneTaq DNA polymerase, NEB) using primers P31 and P32. PCR-amplified donor DNA had approx. concentrations of 50 ng/µl as determined by comparison to DNA marker ladder so that 1 µg of donor DNA (20 µl) was typically employed for each transformation.

Assays to determine plasmid functionality and efficiency were performed as at least biological triplicates. The arithmetic mean was calculated to determine the average number of positive transformants.

### Determining the efficiency of CRISPR/Cas9-mediated mating type switching

*MATa* cells (CEN.PK2-1C or BY4741; *∆trp1*) were loaded with plasmids pMAT (P_HO_-HIS3-T_CYC1_; P_TEF1_-*ALPHA2*-T_TEF1_; *TRP1* marker) and pCAS9id-anti*MATx*/*MATz* (*URA3* marker). Plasmid pMAT mediates tight expression of the *HIS3* selection marker only in *MATα* background (**Fig. S6**) and simultaneously prevents expression from haploid specific promoters (*e.g.*, P_STE2_; P_HO_) in parental *MATa* cells due to the artificial formation of the heterodimeric a1-alpha2 repressor. gRNA-Cas9 complexes encoded by plasmid pCAS9id_anti*MATx*/*MATz* replace the function of the HO endonuclease by mediating DSBs within the X and Z1 region of the *MAT* locus. Plasmid harboring cells were grown overnight in SD-trp-ura medium at 30°C with continuous shaking (200 rpm). Next day, cells were harvested by centrifugation, carefully washed with sterile water, transferred to SGal-trp-ura medium (initial OD_600_ = 0.3) and cultured for 24 h at 30°C to allow for mating type interconversion by initiation of Cas9 expression. Equal numbers of cells (100 µl from suspension with ~ OD_600_ = 1 × 10^-2^ and ~ OD_600_ = 1 × 10^-3^) from respective galactose overnight cultures were plated on YPD and SD-his medium, with the histidine-free medium selecting for cells that were converted from *MATa* to *MATα* (**Fig. S6**). To determine the efficiency of mating type switching, we counted the number of *MATα* cells that were able to survive on SD-his medium and calculated the percentage of cells with switched mating type from all living cells as determined by growth on non-selective YPD medium. Experiments were performed as biological triplicates. Constitutive expression of *ALPHA2* prevents the mating response in *MATa* cells due to the absence of Ste2p, thereby avoiding formation of diploids and autopolyploidization in mixed cultures (galactose medium) which should allow to determine the correct mating type efficiency without risking an underestimation. Switching of mating type was additionally tested for five randomly selected clones by a PCR-based approach as described elsewhere [19].

## RESULTS AND DISCUSSION

### General design of the system

**Figure 1.**
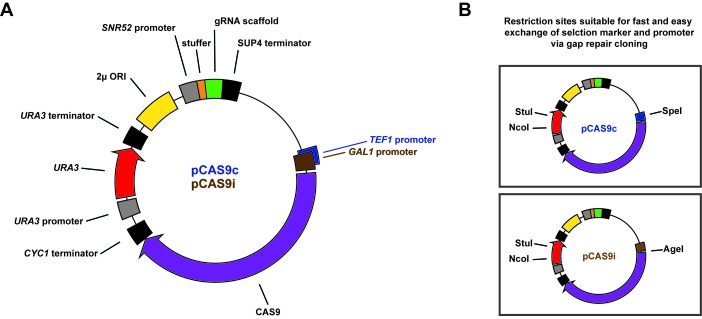
Schematic representation of the all-in-one gRNA-Cas9 expression vectors pCAS9c and pCAS9i. **A.** Both plasmids contain identical backbone elements such as an *URA3* selection marker, a 2-micron (2 µ) sequence and an ampR bacterial selection marker (not shown) as well as an universal gRNA expression cassette consisting of the yeast-endogenous *SNR52* promoter, the constant guide RNA scaffold sequence and the *SUP4* terminator sequence. A stuffer sequence serves as a placeholder for the respective gRNA targeting (protospacer) sequence. Both plasmids differ in the promoter that is driving the expression of the Cas9 gene. Cas9 is either expressed from the constitutive *TEF1* promoter (pCAS9c) or from the inducible *GAL1* promoter (pCAS9i). **B.** Both plasmids harbor specific restriction sites that allow for an easy exchange of the *URA3* selection marker (StuI, NcoI) and the respective promoter sequences (P_TEF1_: SpeI; P_GAL1_: AgeI) *via* gap repair cloning.

We were aiming to develop a CRISPR/Cas9 system for application in *S. cerevisiae* that combines unique advantages and benefits of other existing systems—namely an all-in-one single plasmid based system that especially allows for a simple and convenient introduction of a gRNA protospacer sequence for targeting any desired gene or locus of the yeast (nuclear) genome. For that, we constructed plasmid pCAS9c that harbors a 38 bp stuffer within its gRNA expression cassette which serves as a placeholder for the protospacer sequence to be introduced. The stuffer can be easily removed by simply double-cleaving pCAS9c with KpnI and PmeI (**Fig. 1A**). The “synthesis of DNA fragments in yeast by one-step assembly of overlapping oligonucleotides” [9] was considered to be appropriate as a fast, easy and low-cost method for introducing a desired 20 bp gRNA protospacer sequence into the double-cleaved plasmid. By using this method, protospacer introduction and plasmid circularization would rely solely on the robust yeast-endogenous homologous-recombination (HR) machinery without the need for any PCR applications or any other conventional *in vitro* cloning method. Just commercially available short oligonucleotides that not even need to be hybridized beforehand would be required to determine and introduce any desired protospacer sequence.

Our plasmid pCAS9c was furthermore designed to support its convenient application: Plasmid pCAS9c harbors a 2 µ ORI (**Fig. 1A**) that allows for a high copy number and promotes high expression levels of gRNA and Cas9. Additionally, 2 µ plasmids show decreased segregational stability as compared to centromeric plasmids [20] which is beneficial for removing of pCAS9c after genome editing operations. The *URA3* selection marker allows for positive selection on 5-FOA containing media of cells, which have lost pCAS9c plasmids [21]. However, the application of pCAS9c is not limited to the use of *URA3* as selection marker since pCAS9 plasmids contain unique restriction sites (**Fig. 1B**) that allow for a fast and easy exchange of both, the selection marker as well as the *TEF1* promoter *via* gap repair cloning. This way, our plasmids provide a good basis for generating derivative plasmids harboring dominant antibiotic resistance markers (kanMX, natMX) that are useful to maintain pCAS9 plasmids in wild yeasts and industrial strains that do not support the use of standard selection markers. Promoter exchange might be desired if high Cas9 expression levels from pCAS9c severely impair growth of the background strain, as it was described elsewhere [4].

### Single plasmid-mediated CRISPR/Cas9 genome editing

We selected the *ADE2* locus as target for site-specific cleavage and for subsequent integration of a donor DNA cassette (loxP-*LEU2*-loxP) that serves as a template for homologous recombination-based repair of gRNA-Cas9 induced DNA double-strand breaks (DSBs). The *ADE2* locus was chosen since interruption of the adenine biosynthetic pathway at the stage of Ade2p leads to the intracellular accumulation of a red pigment (AIR) and accordingly to the formation of red colonies [22] which serves as an easy assay read-out. Red colony color in combination with a Leu^+^ phenotype indicates that the desired donor DNA was integrated in the right locus and therefore clearly characterizes positive transformants.

**Figure 2.**
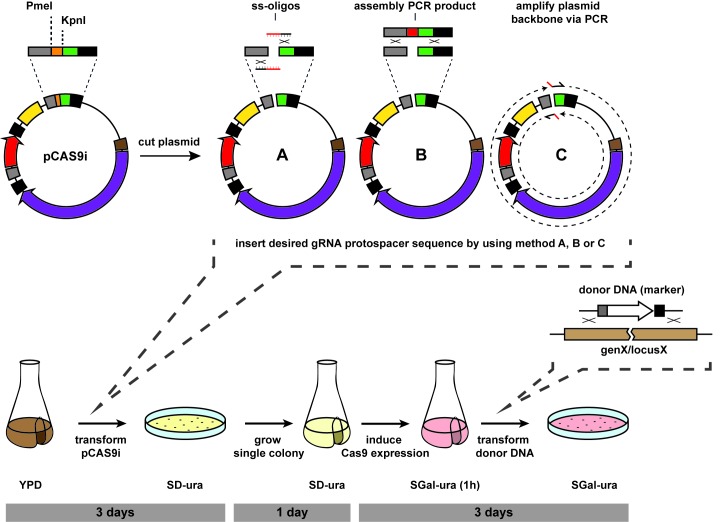
General experimental procedure of pCAS9i-supported CRISPR/Cas9 genome editing in *S. cerevisiae*. The stuffer sequence of plasmid pCAS9i is removed by double-cleaving the vector with KpnI and PmeI (top left). A desired protospacer sequence can be introduced into the linearized vector pCAS9i by method A (one-step assembly of overlapping single-stranded oligonucleotides), method B (introduction of a gRNA expression cassette previously generated with assembly PCR) or method C (PCR amplification of pCAS9i backbone with overhang primers that add the protospacer sequence to both ends of the PCR product) (top row). The target strain is (co-)transformed with all plasmid components so that plasmid assembly and recircularization occurs *in vivo* by recombination-based cloning. Positive transformants are selected on SD-ura agar plates (bottom left). Proper introduction of the desired gRNA protospacer sequence into plasmid pCAS9i can be checked optionally by colony PCR. A single transformant harboring the protospacer containing plasmid (pCAS9i-anti*X*) is grown overnight in SD-ura liquid medium and used for a second transformation with the respective donor DNA to be genomically integrated. Prior to this second transformation pCAS9-anti*X* harboring cells are shifted to SGal-ura medium for 1 h in order to induce Cas9 expression from the *GAL1* promoter and preloading cells with gRNA and the Cas9 endonuclease. Transformed cells can be selected on appropriate SGal agar medium. If a donor DNA contains a selection marker, cells may be spread on SGal-ura medium that is lacking for a second nutrient (bottom row).

**Figure 3.**
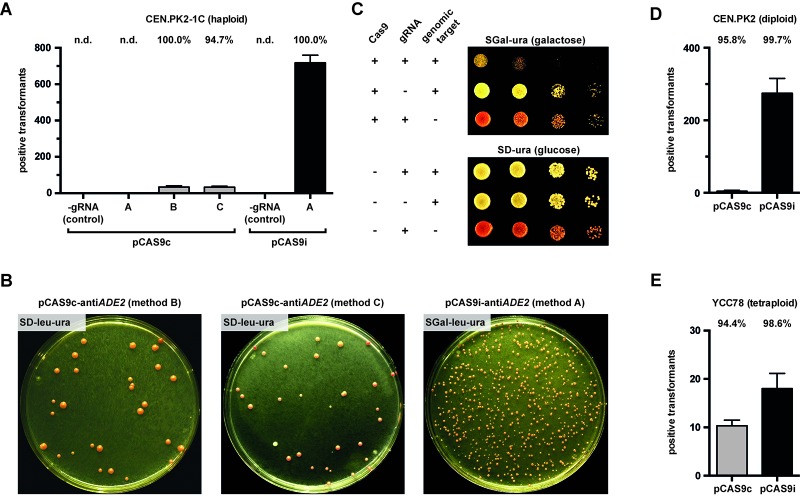
Application of Cas9- and gRNA-encoding plasmids pCAS9c- and pCAS9i for CRISPR/Cas9-supported genome editing of yeast cells with different ploidy levels. **A.** An *ADE2* disruption (*ade2*::loxP-*LEU2*-loxP) was introduced in the haploid strain CEN.PK2-1C by pCAS9c- (grey bars) or pCAS9i- (black bars) based approaches and the number of positive (knockout) transformants was determined. gRNA negative controls (-gRNA; used unmodified plasmids pCAS9c or pCAS9i that do not encode for a functional anti-*ADE2* gRNA) yielded no positive transformants. Mean values and standard deviations from at least triplicate experiments are indicated. The identical number of cells was employed for each transformation and approach. Efficiencies are indicated above the bars and represent the percentage of positive transformants of all cells that survived after transformation on the SGal-leu-ura selection media. n.d.: not detected. **B.** Representative agar plates showing the number of positive *ADE2* knockout transformants of the haploid strain CEN.PK2-1C (see A) that were yielded with approaches that used pCAS9c (method B or method C) or pCAS9i (method A). Cells were selected on indicated media, respectively. **C.** pCAS9i-mediated toxicity was investigated by spotting plasmid pCAS9i-anti*ADE2* harboring cells in 10-fold serial dilutions on glucose and galactose containing media, respectively. Growth on galactose containing media was severely impaired as Cas9 expression is activated. Control strains that do not express a functional gRNA (empty pCAS9i without anti-*ADE2* protospacer sequence) or that do not provide a proper genomic protospacer sequence (cells are already disrupted in *ADE2* by previously CRISPR/Cas9-supported donor DNA integration) grew unimpaired on galactose containing medium. **D.** Number of diploid CEN.PK2 cells with successfully disrupted *ADE2* by pCAS9c- (grey bars) or pCAS9i- (black bars) based approaches. **E.** For the tetraploid strain YCC78, CRISPR/Cas9-supported disruption of *ADE8* (*ade8*::loxP-kanMX-loxP) was performed with a pCAS9c- or a pCAS9i-based approach and the number of positive transformants was determined. Unless specified differently (as for CEN.PK2-1C), anti-*ADE2*/*ADE8* protospacer sequence was introduced into pCAS9c by method C and into pCAS9i by method A. For further explanations of the graphs see (A).

To functionally test our plasmid construct pCAS9c, we co-transformed cells of the haploid laboratory strain CEN.PK2-1C with KpnI & PmeI cleaved pCAS9c, oligonucleotides carrying the *ADE2* targeting protospacer sequence or the complementary sequence, respectively, at their 5’ ends (method A) (**Fig. 2**) as well as PCR-amplified loxP-*LEU2*-loxP donor DNA. Method A would serve as an extraordinarily straightforward approach if protospacer introduction into plasmid pCAS9c by assembling overlapping oligonucleotides would be efficient enough to allow for a single transformation to deliver all the required CRISPR/Cas9 components (Cas9, gRNA, donor DNA) into a cell. However, no transformants could be obtained by using this approach (**Fig. 3A**) which might be due to a low efficiency of recombination between two individual ss-oligonucleotides and the linearized plasmid compared to when a single dsDNA fragment derived from two annealed ss-oligos is employed as protospacer insert that worked for other approaches [2]. To test the general functionality of plasmid pCAS9c, we repeated the previous transformation, but replaced both ss-oligonucleotides with a pre-assembled gRNA expression cassette (10 µl) (method B) constructed by assembly PCR (**Fig. S1**). This approach gave approximately 30 positive transformants, whereas a control experiment without gRNA in which only uncleaved and unmodified pCAS9c was used (-gRNA control) yielded no transformants (**Fig. 3A** and **3B**). This demonstrates that anti-*ADE2* gRNA and Cas9 were successfully expressed from pCAS9c and CRISPR/Cas9-mediated cleavage of *ADE2* efficiently promoted genomic integration of the loxP-*LEU2*-loxP donor DNA. We further tested, whether our plasmid pCAS9c is also suitable for an approach where the gRNA protospacer sequence is introduced by amplifying the whole vector backbone with overhang primers carrying the anti-*ADE2* protospacer sequence at their 5’ ends, respectively (method C). Circularization of the plasmid and thereby reconstitution of the functional gRNA expression cassette are obtained by *in vivo* homologous recombination. CEN.PK2-1C was co-transformed with the PCR-amplified pCAS9c backbone and loxP-*LEU2*-loxP donor DNA. This approach yielded a similar number of transformants as the previously described approach that used a pre-assembled gRNA expression cassette (**Fig. 3A** and **3B**).

### High-efficient genome editing by preloading yeast with all-in-one CRISPR/Cas9 vectors

To follow up on a straightforward approach that utilizes one-step assembly of overlapping single-stranded oligonucleotides for introducing the protospacer sequence, we modified plasmid pCAS9c by replacing its constitutive *TEF1* promoter with the tightly controllable and inducible yeast endogenous *GAL1* promoter, yielding plasmid pCAS9i (**Fig. 1A**). A desired protospacer sequence can be introduced into pCAS9i *via in vivo* homologous recombination-based cloning by either using cloning method A, B or C without being at risk to generate lethal gRNA-Cas9 induced DNA DSBs. This way, plasmid assembly and CRISPR/Cas9-mediated genome editing (*e.g.*, donor DNA integration) can be chronologically separated into two individual sequential steps.

Cloning methods A, B and C (**Fig. 2**) were proved to be suitable to introduce the *ADE2* gRNA targeting sequence into plasmid pCAS9i as confirmed by colony PCR (**Fig. S2**). Since cloning method A is the least labor-intensive and therefore most interesting approach, we only proceeded with three randomly selected transformants generated with this method. All cells derived from a single transformant harboring plasmid pCAS9i that encodes for the Cas9 endonuclease as well as the anti-*ADE2* gRNA. By switching cells to galactose medium (SGal-ura) for 1 h prior to transformation with donor DNA (**Fig. 2**), every single cell that is used for transformation should be already pre-loaded with anti-*ADE2* gRNA and Cas9, so that a functional CRISPR/Cas9 system is constituted, enabling introduction of DSBs in the *ADE2* locus. Only those cells in which DSBs were repaired by correct integration of the loxP-*LEU2*-loxP cassette were selected on appropriate medium (SGal-leu-ura). We expected the pCAS9i-based approach to be much more efficient than the one that uses pCAS9c, since negative effects resulting from the inevitable requirement of co-transforming more than one system component, as well as of temporal timing of gRNA/Cas9 expression and donor DNA delivery/ availability are not prejudicial to the overall efficiency anymore. Indeed, with an identical number of cells employed for transformation, we obtained with the pCAS9i-based approach some 20-fold more positive transformants, as compared to the afore described approaches (**Fig. 3A** and **3B**). The obtained transformation efficiencies (**Table 2**) were in the same range as for previously reported CRISPR/Cas9 applications [15]. Identical results can be expected when strains are used that were derived from cloning methods B and C since both of them yield exactly the same plasmid pCAS9i-anti*ADE2*. A control strain harboring unmodified pCAS9i (without the anti-*ADE2* protospacer sequence) yielded no positive transformants after transformation with donor DNA (**Fig. 3A**).

Disruption of *ADE2* by genomic integration of the loxP-*LEU2*-loxP cassette was confirmed by colony PCR with five randomly selected transformants obtained by using plasmids pCAS9c and pCAS9i-based approaches. All tested transformants were confirmed to be positive with no exception (**Fig. S3**).

To characterize the efficiency of plasmid loss after genome editing, positive transformants were cultured for a single round in non-selective medium (YPD) overnight. Subsequently, cells were spread on YPD to obtain single colonies and colonies were picked and streaked on YPD and SD-ura agar plates. Ninety-four percent (47 from 50) of the cells did not grow on uracil-free medium anymore which indicates a high frequency of plasmid loss. In daily lab applications, *URA3* would also allow for a positive selection of plasmid-freed cells on 5-FOA containing medium.

### pCAS9i-mediated toxicity

Unrepaired DNA DSBs are lethal in *S. cerevisiae* [23,24] leading to a beneficial reduction of false positive clones in CRISPR/Cas9 applications. We wanted to determine the degree of toxicity caused by the expression of a functional CRISPR/Cas9 system from plasmid pCAS9i-anti*ADE2*. For that, we spotted plasmid pCAS9i-anti*ADE2* harboring cells in 10-fold serial dilutions on uracil-free glucose (SD-ura) or galactose (SGal-ura) containing medium that only selects for plasmid-carrying cells. On glucose containing medium, cells grew unaffected as Cas9 expression is repressed, whereas galactose containing medium, *i.e.* expression of a functional Cas9-gRNA complex, strongly impaired growth (**Fig. 3C**). As controls, we spotted cells that were either expressing a functional *ADE2*-targeting CRISPR/Cas9 system but provided no proper genomic target sequence that could be addressed by the Cas9-gRNA complex (a strain that was already disrupted in *ADE2* by previously CRISPR/Cas9-supported donor DNA integration) or we employed a control strain that contained the native *ADE2* locus but expressed no *ADE2*-targeting gRNA (empty pCAS9i harboring the protospacer stuffer sequence). Both strains grew unimpaired on galactose containing media (**Fig. 3C**). These results indicate a high degree of CRISPR/Cas9-induced toxicity when no donor DNA for HR-mediated repair of DSBs is available and helps to select for positive transformants in our pCAS9i-based CRISPR/Cas9 approach.

For a similar test, we plated highly diluted suspensions of pCAS9i-anti*ADE2* harboring CEN.PK2-1C cells as well as of the previously described control strains on glucose- (SD-ura) and galactose containing (SGal-ura) media to obtain single colonies. On glucose containing medium, colonies recovered from all plated cells for all tested strains (**Fig. S4**), whereas galactose strongly impaired the appearance of colonies when pCAS9i-anti*ADE2* was present in an unmodified CEN.PK2-1C genetic background (**Fig. S4A**). Only a small percentage of cells (2.7% ± 0.5%; *n* = 3) formed colonies with the same size as cells did on the reference media (SD-ura) (**Fig. S4A**). The vast majority (93.3% ± 2.5%; *n* = 3) of these cells that survived and grew unaffected on the SGal-ura medium turned out to be *ade2* knockout mutants as evaluated from their phenotype (red colony color; Ade^-^ as confirmed by replica plating on SGal-ura-ade medium). *ADE2* knockouts most probably resulted from NHEJ-mediated imperfect DNA repair and accompanying indel errors. Those errors additionally destroy the proper genomic gRNA-Cas9 targeting sequence thus protecting cells from further toxic DNA DSBs and simultaneously selecting for cells with imprecisely repaired DNA. This way, our pCAS9i-based approach provides an efficient tool to introduce gene knockouts that is extremely simple in application, just requires two approximately 50 bp oligonucleotides and not even involves a single PCR step.

### Editing the genome of diploid and tetraploid yeast strains

Unlike most laboratory strains, wild yeasts and industrial strains do not harbor a single set of chromosomes, but they are rather diploid [25-27] or even feature higher ploidy levels that can *e.g.*, derive from allo- or autopolyploidization [28]. Since metabolic engineering approaches to improve fermentation performance or product yield of industrial strains often require the knockout of genes that are *e.g.*, involved in competing metabolic pathways [29], highly efficient CRISPR/Cas9 systems are crucial as multiple copies of the same gene have to be addressed at once. Wild yeasts are usually not accessible to metabolic engineering approaches since they do not feature any auxotrophies [25] and therefore *e.g.*, do not support plasmid maintenance. In this case, the deletion of all copies of a marker gene is required to generate auxotrophic mutants [30]. To test our plasmid-based systems for such purposes, we employed the diploid (2 *n*) strain CEN.PK2 as well as the tetraploid (4 *n*) strain YCC78 instead of the haploid strain CEN.PK2-1C. For diploid CEN.PK2, *ADE2* was targeted the same way as previously described for its haploid strain derivative with the difference that red coloring of colonies this time indicates the knockout of both *ADE2* copies. However, the *ADE2*-based assay could not be applied for the tetraploid strain YCC78 since its four *ADE2* copies already encode for a dysfunctional protein variant (**Table 1**). Here, we targeted *ADE8* instead, which encodes for a protein that acts upstream of Ade2p in the adenine biosynthetic pathway and exploited the epistatic relationship of *ade8* over *ade2* to select for white colonies [22]. White coloring of colonies indicates a knockout of all four *ADE8* gene copies.

For application in strain backgrounds with higher ploidy levels, we introduced the respective protospacer sequences into plasmid pCAS9c with method C and into plasmid pCAS9i with method A (**Fig. 2**). By employing plasmid pCAS9c for disrupting *ADE2* in diploid CEN.PK2 cells, we obtained only a few positive transformants, whereas the pCAS9i-based approach yielded hundreds of transformants (**Fig. 3D**) that could be evaluated as being positive based on their phenotype (red colony color; Leu^+^). Also for the tetraploid background strain, both plasmid-based approaches yielded quadruple *ade8* knockout mutants, as clearly evident from the white appearance of the obtained colonies. Moreover, successful genomic integration of the loxP-kanMX-loxP donor DNA into the *ADE8* locus was confirmed by colony PCR (**Fig. S5**). The pCAS9i-approach yielded twice as many positive transformants of YCC78 as compared to the pCAS9c use, even though the absolute number of positive transformants was generally lower than for haploid or diploid strain backgrounds (**Fig. 3D**). Measured transformation efficiencies emphasize highly efficient genome editing (**Table 2**). A few single di- and tetraploid transformants appeared to feature the respective positive growth phenotype (Leu^+^ or G418 resistant), indicating integration of the marker cassette, but seem not to have all gene copies deleted as indicated by their colony coloring, *e.g.*, white diploids or red tetraploids. Such false transformants arose at lower rate for pCAS9i- (2 *n*: < 1%; 4 *n*: < 2%) than for pCAS9c-based (2 *n*: < 5% & 4 *n*: < 6%) approaches.

### Multiplex genome editing

**Figure 4.**
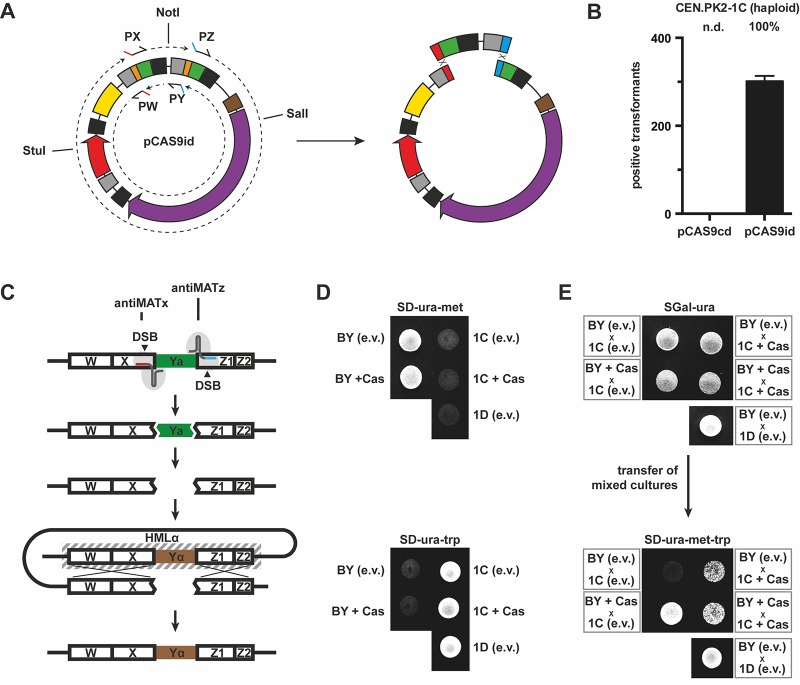
Multiplex genome editing and CRISPR/Cas9-mediated mating type switching. **A.** Plasmid pCAS9id is amplified by two individual PCRs to add the two different protospacer sequences to one end of the two fragments, respectively. Linearized plasmids are employed as PCR template to avoid the amplification of the undesired plasmid fragment (NotI-cleaved plasmid for use with primers PW & PZ; StuI/SalI-cleaved plasmid for use with primers PX & PY). Note: Primers PX & PZ and PW & PY, respectively, contain identical annealing sequences which would result in four different PCR products if uncut plasmid is employed as PCR template. The gRNA protospacer containing plasmid is subsequently circularized by homologous recombination *in vivo*. **B.** Simultaneous disruption of *ADE2* (*ade2*::loxP-*LEU2*-loxP) and *CAN1* (*can1*::*HIS3*) in the haploid strain CEN.PK2-1C was successfully achieved by using an pCAS9id-based approach. The efficiency is indicated above the bar and represents the percentage of positive transformants of all cells that survived on SGal-leu-ura-his-arg + canavanine (60 mg/L) selection medium after transformation. **C.** The schematic drawing shows the organization of the mating type (*MAT*) locus containing the constant W, X, Z1 and Z2 regions as well as the variable mating type specific Y regions. Plasmid pCAS9id-anti*MATx*/*MATz* encodes for two different gRNAs targeting the X and Z1 region, respectively. Cas9-mediated DSBs completely excise the Y region resulting in a gap within the *MAT* locus that can be repaired by HDR. Hidden *MAT* loci can be used as donor templates for HDR thus leading to mating type switching. **D.** Prototrophies (Met^+^ and Trp^+^) of haploid strains used for mating assays is demonstrated. **E.** Generation of diploids was tested by transferring mixed cultures to SD-ura-met-trp medium that selects for mating-induced combination of unique prototrophies originating from one of the haploid strains, respectively. Mating assays demonstrate that CRISPR/Cas9-mediated DSBs within the *MAT* locus successfully lead to mating type switching in CEN.PK2-1C (1C; *MATa*) and BY4741 (BY, *MATa*). Generation of diploids in a mixed culture (X) was only possible when at least one of the two strains expressed Cas9 from plasmid pCAS9id-*MATx*/*MATz* (+Cas) on galactose medium (SGal-ura). Empty vectors (e.v.) did not support mating type switching.

To test our plasmid systems for multiplex genome editing applications, we inserted a second gRNA expression cassette into plasmids pCAS9c and pCAS9i yielding plasmids pCAS9cd and pCAS9id (duplex) that allow for expression of two different gRNAs. Each plasmid can be amplified by two individual PCRs that add different protospacer sequences to one end of each fragment, respectively (similar to method C). These protospacer sequences function additionally as homology sequences for correct plasmid circularization by homologous recombination *in vivo*. Since both pairs of primers feature identical annealing sequences, NotI-digested (Primers PW & PZ; for large fragment) or StuI/SalI-cleaved (Primers PX & PY; for short fragment) plasmids have to be employed as PCR templates to prevent the amplification of the corresponding undesired part of the plasmid (**Fig. 4A**). We chose the *CAN1* locus as second genomic target since its disruption allows for positive selection and therefore provides the possibility to select for the correct integration of the right donor DNA into the right locus as it is also true for the previously used *ADE2*-based assay.

We co-transformed haploid CEN.PK2-1C cells with both pCAS9cd-anti*ADE2*/*CAN1* PCR fragments as well as with both donor DNA cassettes (loxP-*LEU2*-loxP for *ADE2* and *HIS3* for *CAN1*) and plated cells on SD-ura-leu-his-arg + canavanine medium. However, no transformants could be obtained. In contrast, cells that were preloaded with pCAS9id-anti*ADE2*/*CAN1* and subsequently transformed with both donor DNA fragments were successfully deleted in both genes simultaneously with high efficiency (**Fig. 4B**). We obtained approximately 300 positive transformants as one could evaluate from their phenotype (red colony color; canavanine resistant; Leu^+^; His^+^) thereby also confirming the functionality of pCAS9id.

### CRISPR/Cas9-mediated mating type switching of yeast

Induction of a double strand break followed by homologous recombination-based repair of the cleaved and subsequently partially degraded genomic DNA is the core part of the inherent capacity of homothallic yeast cells to switch from one mating type to the other. However, most laboratory strains are unable to switch their mating type because they either do not express a (functional) HO endonuclease [31] or feature so-called stuck mutations [32] within the native recognition sequence of the site-specific HO endonuclease. Whereas the first mentioned limitation can be simply overcome by *e.g.*, plasmid-based expression of native *HO* [33], the latter case poses a more severe problem when mating type switching is desired. Our inducible double-cleaving CRISPR/Cas9 system was tested to replace the native function of *HO* in ∆*ho* strains. Thus, mating type switching in genetic backgrounds featuring stuck mutations within the *MAT* locus (such as in S288c derived strains) should be enabled due to the easy adaptability of CRISPR/Cas9 cleavage sites.

To induce double-strand breaks within the *MAT* locus by using our already tested multiplex vector pCAS9id (see above), two different protospacer sequences were selected that address sequences of the invariable X (anti*MATx*) and Z1 (anti*MATz*) regions present in both *MAT* alleles, thereby mediating a complete excision of the respective Y region (**Fig. 4C**). To test if the resulting gap within the *MAT* locus is repaired by the endogenous *HDR* using *HMLα* or *HMRa* as donor sequences, a mating assay was performed. For that, two different strains featuring the same mating type, BY4741 (*MATa*) and CEN.PK2-1C (*MATa*), thus being unable to mate with each other, were employed. Each of both strains possesses an auxotrophic mutation which does not apply to the other, thus featuring a unique growth phenotype, respectively*–*BY4741 grows on medium lacking methionine (-met) (**Fig. 4D**, top) whereas CEN.PK2-1C grows on medium without tryptophan (-trp) (**Fig. 4D**, bottom). The generation of diploids from both different strains requires mating type switching in at least one of both strains. To test for a successful CRISPR/Cas9-mediated mating type switching, mixtures of both strains harboring either pCAS9id-anti*MATx*/*MATz* or an empty *URA3* vector (*e.v.*, pGREG506 [18]; unmodified pCAS9id was not used for empty vector controls to prevent artifacts that might result from the simultaneous presence of two different pCAS9id species in diploid cells) were plated on medium that selects for both plasmids and supports plasmid-based expression of Cas9 were applicable (SGal-ura) (**Fig. 4E**, top). Resulting diploids are expected to be subsequently selected on SD-ura-met-trp triple drop-out medium (**Fig. 4E**, bottom). Indeed, diploids resulted from all combinations involving at least one strain that harbors a functional pCAS9id-anti*MATx*/*MATz*, thus clearly indicating that CRISPR/Cas9 induced DSB within the *MAT* locus can result in successful mating type switching. A negative control that used strains harboring empty vectors did not yield diploids whereas the positive control that used strains already featuring different mating types, BY4741 (*MATa*) and CEN.PK2-1D (*MATα*), led to mating and formation of diploids, as expected. Inducing DSB by CRISPR/Cas9 also helped to bypass impaired HO-mediated *MAT* cleavage due to stuck mutations as demonstrated by successful switching the mating type of S288c-derived BY4741. For determining the efficiency of mating type interconversion, we exploited mating type-specific expression of the *HIS3* selection marker to easily select for cells that switched from *MATa* to *MATα* by a pure growth phenotype (**Fig. S6**). Mating type conversion occurred for 5.1% ± 0.7% of pCAS9id-anti*MATx*/*MATz* harboring CEN.PK2-1C cells grown in galactose medium and for 2.0% ± 0.8% of BY4741 cells under identical conditions. Five randomly selected clones were checked exemplarily for switched mating type by colony PCR (**Fig. S7**). However, it should be noted that our approach does not trap cells in *MATα* and re-switching to the initial mating type is possible as it is also true for plasmid-based expression of native *HO*. Nevertheless, the obtained efficiencies are in the same range as that recently published [34] from a similar approach and exceeded the previously reported CRISPR/Cas9-supported transformation efficiencies (**Table 2**) ~1000-fold. These results clearly demonstrate that CRISPR/Cas9-mediated DSBs can also be repaired with high efficiency by using genomically encoded donor DNA for HDR without a need for external linear donor DNA elements and further emphasize the practical applicability of our inducible CRISPR/Cas9 all-in-one vectors.

In conclusion, here we introduced a new set of all-in-one CRISPR/Cas9 plasmids that allow for a simple and convenient application of the technology in *S. cerevisiae* by combining beneficial features of different existing approaches in one system. The inducible CRISPR/Cas9 systems presented here can easily be integrated into established workflows for marker-based genome editing approaches that are commonly used in the community or used for CRISPR-interference/activation applications. Furthermore, cells once preloaded with our inducible CRISPR/Cas9 vectors can serve as a universal platform for applications were genomic integrations of different DNA fragments or even whole DNA libraries into the same locus is required. The easy architecture of our plasmids that has been disclosed in detail moreover allows to tailor our vectors for individual personal requirements *e.g.*, by changing the selectable marker or the promoter driving Cas9 expression. Easy and simple Cas9 nickase applications would be furthermore enabled by introducing the nCAS9 (nicking) encoding sequence into our successfully tested all-in-one multiplex vectors. Our homologous recombination based cloning approaches are moreover compatible with *in vivo* cloning methods but are not limited to it and can be combined with commercially available HR-based kits for DNA assembly *in vitro* such as Gibson Assembly or related methods.

## Supplementary Material

Supplementary information **Figure S1.** Gel-electrophoretic analysis of assembly PCR intermediates and the final assembly product.
**Figure S2.** Colony PCR products for confirming proper introduction of anti-*ADE2* protospacer sequence in plasmid pCAS9i analyzed by agarose gel electrophoresis.
**Figure S3.** Colony PCR products for confirming the disruption of *ADE2* by integration of loxP-*LEU2*-loxP donor DNA in an CEN.PK2-1C background analyzed by agarose gel electrophoresis.
**Figure S4.** A functional *ADE2*-targeting CRISPR/Cas9 system expressed from pCAS9i is toxic for yeast.
**Figure S5.** Colony PCR products for confirming the disruption of *ADE8* by integration of loxP-kanMX-loxP donor DNA in an YCC78 background analyzed by agarose gel electrophoresis.
**Figure S6.** pMAT harboring *MATα* cells functionally express *HIS3*.
**Figure S7.** Colony PCR products for confirming CRISPR/Cas9-mediated mating type switching from *MATa* (CEN.PK2-1C) to *MATα* (CEN.PK2-1D) analyzed by agarose gel electrophoresis.
**Table S1**. Primers and oligonucleotides used in this study.
Supplementary information of this article can be found online at
http://www.jbmethods.org/jbm/rt/suppFiles/254.

## Figures and Tables

**Table 1.** Yeast strains used in this study.

Strain	Genotype	Reference
BY4741	*MATa; his3∆1; leu2∆0*; *met15∆0*; *ura3∆0*	[10]
BY4741; ∆*trp1*	*BY4741*; *trp1::kanMX4*	[13]
CEN.PK2-1C	*MATa; his3∆1*; *leu2-3*,112; *ura3-52*; *trp1-289*; *MAL2-8c*; *SUC2*	[11]
CEN.PK2-1D	*MATα; his3∆1*; *leu2-3*,112; *ura3-52*; *trp1-289*; *MAL2*-*8c*; *SUC2*	[11]
CEN.PK2	*MATa/ MATα*; *his3∆1*/ *his3∆1*; *leu2-3*,112/ *leu2-3*,112; *ura3-52*/ *ura3-52*; *trp1-289*/ *trp1-289*; *MAL2-8c*/ *MAL2-8c*; *SUC2*/ *SUC2*	[11]
YCC78 (Y558)	*MATa/ MATa*/ *MATα*/ *MATα*; *ura3-52*/ *ura3-52*/ *ura3-52*/ *ura3-52;* *LYS2/ lys2-801*/ *LYS2*/ *lys2-801;* *ade2-101*/ *ade2-101*/ *ade2-101*/ *ade2-101;* *HIS3/ his3∆200*/ *his3∆200*/ *his3∆200;* *trp1∆1/ trp1∆1*/ *trp1∆1*/ *trp1∆1;* *TYR1/ tyr1*/ *TYR1*/ *TYR1*	[12]

**Table 2.** Transformation efficiencies obtained for pCAS9i(d)-supported gene editing.

Strain	Ploidy	Plasmid	Transformation efficiency^a^
CEN.PK2-1C	1 *n*	pCAS9i-anti*ADE2*	2.8 ± 0.6 × 10^-5^
CEN.PK2-1C	1 *n*	pCAS9id-anti*ADE2/CAN1*	1.2 ± 0.2 × 10^-5^
CEN-PK2	2 *n*	pCAS9i-anti*ADE2*	2.1 ± 0.5 × 10^-5^
YCC78	4 *n*	pCAS9i-anti*ADE8*	3.6 ± 1.6 × 10^-6^

^a^Transformation efficiency values were defined as the ratio of positive transformants to all viable cells that were initially employed for transformation (*n* = 3).
